# The role of non-operative management (NOM) in perforated diverticulitis: a systematic review

**DOI:** 10.1007/s00423-025-03937-9

**Published:** 2026-01-16

**Authors:** Roberto Cirocchi, Matteo Matteucci, Giulio Maria Mari, Michelangelo Campanale, Gabrio Bassotti, Justin Davies, Mauro Zago, Antonio Pesce, Bruno Cirillo, Gioia Brachini, Andrea Mingoli, Riccardo Nascimbeni

**Affiliations:** 1 Department of Medicine and Surgery, University of Perugia, Perugia, Italy; 2 Department of Medicine and General Surgery, University of Milan, Milan, Italy; 3 Department of General Surgery, Ospedale Pio XI, Desio, Italy; 4 Department of Gatroenterology, University of Perugia, Perugia, Italy; 5 Cambridge Colorectal Unit, Addenbrooke’s Hospital, Cambridge University Hospitals NHS Foundation Trust, Cambridge, UK; 6 Department of General Surgery, A. Manzoni Hospital, Lecco, Italy; 7 Department of Surgery, AUSL of Ferrara, Ferrara, Italy; 8 Department of Surgery, Sapienza University, Rome, Italy; 9 Department of Molecular and Translational Medicine, University of Brescia, Brescia, Italy

**Keywords:** Diverticulitis, Complicated diverticulitis, Free air, Pericolic air, Distant air, Non- operative management

## Abstract

**Background:**

One of the most common and significant complication of acute diverticulitis is visceral perforation. Current clinical guidelines suggest conservative medical therapy can be adopted for selected patients with perforation, especially those with pericolic air, while its role remains less clear in cases of distant air. The aim of our study is to evaluate the role of non-operative management (NOM) in case of pericolic and distant air.

**Materials and methods:**

The authors conducted a comprehensive literature review; this search yielded 23 studies (17 retrospective, 5 prospective and 1 randomized control trial), including 2689 patients.

**Results:**

Conservative management of patients with air in perforated diverticulitis was safe and feasible, with a overall pooled success rate of 90.2% (95% CI: 86.4–93). Specifically, among patients with pericolic extraluminal air, the success rate of NOM was 89.9%. In contrast, the role of NOM in cases with distant free air remains uncertain, with a lower success rate of only 27.8%.

**Conclusion:**

Non-operative management (NOM) appears safe and effective for patients with perforated diverticulitis and pericolic extraluminal air, provided there are no clinical signs of generalized peritonitis. In contrast, its role in cases with distant free air is highly uncertain: the pooled success rate is lower, even among hemodynamically stable patients. Based on these findings, early surgical management should be strongly considered for patients with distant free air, while NOM should only be attempted in highly selected cases under strict clinical and radiological monitoring. Conversely, NOM can be confidently recommended for patients with pericolic air who are stable and without diffuse peritonitis.

## Introduction

Visceral perforation is a significant complication of acute diverticulitis, involving the escape of air and intestinal contents into the peritoneal cavity. The presence of free air is referred to as pneumoperitoneum and free air can be classified as pericolic air or distant air. According to World Society of Emergency Surgery (WSES) classification [1], complicated acute diverticulitis can be divided into 4 stages.

Abdominal CT scan is the first-line diagnostic tool for suspected acute diverticulitis, [2, 3]; in fact abdominal CT scan has greater sensitivity for detecting free air from colonic perforation, including small amounts (pericolic air bubbles) that are often missed on a direct abdominal X-ray. Approximately 64% of patients with colon perforation present with extraluminal air on an emergency CT scan [4]. The increasing use and high sensitivity of CT scans have introduced a new question regarding the treatment of patients with extraluminal air without signs of peritonitis: when is conservative treatment sufficient, and when is emergency surgery necessary?

Current clinical practice suggests that conservative medical therapy is appropriate for patients with pericolic air without signs of peritonitis, whereas this approach is less common for cases with distant air [1]. Our research aimed to systematically review the literature on the treatment of acute perforated diverticulitis with extraluminal air, focusing on the feasibility and success rate of conservative treatment for pericolic or distant air.

## Materials and methods

The study protocol for this systematic review was registered as CRD42024603940 in PROSPERO (http://www.crd.york.ac.uk/prospero). A literature search was conducted up to 1 December 2024, following the Preferred Reporting Items for Systematic Reviews and Meta-Analyses guidelines. The review was conducted in accordance with the PRISMA 2020 guidelines, and the study selection process is illustrated in the PRISMA flow diagram [5].

### Search methods for identification of studies

We conducted a systematic literature search to identify studies reporting outcomes of non-operative management of perforated colonic diverticulitis with CT-confirmed extraluminal air. Electronic databases searched were PubMed (MEDLINE), Scopus, Web of Science, and Cochrane CENTRAL. No limits were applied on language, country, or publication year. Searches were performed on 1 December 2024. Retrieved records from all databases were exported to EndNote (or an equivalent reference manager) and deduplicated prior to screening.

## Data collection and analysis

Two reviewers (R.C. and M.M.) independently screened titles/abstracts and assessed full texts; disagreements were resolved by discussion and, when necessary, by a third reviewer. We also performed reference list screening and citation tracking to identify additional records. The full, reproducible search strategies for each database (including all query strings, fields, and operators) are provided in Supplementary Appendix 1. 

## Inclusion and exclusion criteria

Free intraperitoneal air was categorized as pericolic (WSES IA) or distant air (WSES IIB). A standardized definition of pericolic extraluminal air was not used in the search, and retroperitoneal air was reported as a distinct group. Only studies that examined and reported outcomes of patients with colonic diverticulitis complicated by perforation with the presence of extraluminal air confirmed by CT were considered eligible.

The inclusion criteria for the selected studies included:

- Presence of extraluminal air during an episode of acute perforated diverticulitis documented by CT scan.
- A priori we defined failure as emergency surgery; however, because some studies count percutaneous drain (PD) as failure, we performed subgroup analyses. One of the major sources of heterogeneity across the included studies is the operational definition of “failure” in non-operative management (NOM). While our primary analysis defined failure as the need for emergency surgery, several studies also considered percutaneous drainage (PD) as a failure event. This definitional variability introduces bias in pooled success rates and must be explicitly addressed. We adopted an operational primary definition of “failure” as the need for emergency surgical intervention. To assess the impact of definitional variability across included studies, we extracted each study’s original operational definition of failure and categorised them a priori as: (1) “surgery only”; (2) “surgery or percutaneous drainage”; (3) “any invasive intervention/not specified”. Extraction was performed independently by two reviewers; disagreements were resolved by discussion with a third reviewer. To account for this, we extracted in Table 2 the original operational definition of “failure” from each included study and recorded it in a dedicated column of the study table. Definitions were classified a priori into three categories: (1) “surgery only”; (2) “surgery or percutaneous drainage (PD)”; (3) “any invasive intervention/not specified” (reported as NR when not stated). For subgroup analyses, studies were then pooled into two mutually exclusive groups: Group A — PD counted as failure (PD = failure); Group B — PD considered part of successful conservative management (PD ≠ failure). We performed descriptive comparisons of PD utilization and NOM success rates between these two groups. For quantitative syntheses we performed meta-analyses of proportions using a random-effects model (REML), reporting 95% confidence intervals and heterogeneity statistics (I²). Predefined subgroup analyses were performed by failure definition, and sensitivity analyses were planned excluding large studies and restricting to prospective cohorts.

Abstracts, posters, letters to the editor, and case reports were excluded. Additionally, studies reporting fewer than 10 patients with study clusters were excluded. Data collection and results were summarized in tables.

### Data extraction

Data extracted from each study included the country and setting where it was conducted, the study design (observational retrospective or prospective), the enrolment period, the follow-up period, the definition of pericolic extraluminal air, patient selection criteria, the colonic segment, and the type of antibiotics administered. For each reported outcome we extracted the number of patients contributing to that outcome (per-study numerator and denominator). When an outcome was not reported in a given study the study was excluded from the denominator for that outcome; we did not impute missing outcome data. Per-outcome denominators are therefore outcome-specific and may differ from the total pooled population.

Outcome denominators vary because reporting across primary studies was incomplete and heterogeneous. To avoid biased imputations we used study-specific denominators for each outcome (complete-case analysis): each pooled estimate includes only patients from studies that explicitly reported the relevant outcome. Analyses were performed in R (R Foundation for Statistical Computing); meta‑analyses were conducted with the meta for package.

### Assessment of risk of bias

Risk of bias was assessed at study level using the recommended tools for each study design. For randomized trials we applied the RoB 2 tool (revised Cochrane risk-of-bias tool for randomized trials) [29]and for non-randomized observational studies we applied ROBINS-I (Risk Of Bias In Non-randomised Studies — of Interventions) [30]. Two reviewers (R.C. and M.M.) independently judged risk of bias for each study; disagreements were resolved by discussion and, when necessary, by a third reviewer (R.N.). For each study we rated all ROBINS-I domains as Low, Moderate, Serious or Critical and all RoB 2 domains as Low, Some Concerns or High.

### Statistical methods

We performed meta-analyses of proportions using a random-effects model estimated with restricted maximum likelihood (REML) on the logit scale. Studies with zero events were handled with a continuity correction of 0.5. Pooled proportions were back-transformed to the original scale and reported with 95% confidence intervals. Between-study heterogeneity was quantified with the I² statistic. Predefined subgroup analyses were performed for location of free air (pericolic vs. distant) and for operational definition of failure (PD counted as failure vs. PD not counted as failure). Sensitivity analyses included exclusion of very large studies and restriction to prospective cohorts. All analyses were conducted in R using the metafor package; corresponding forest plots for the overall population and subgroups are provided.

## Results

### Literature search

**Fig. 1:**
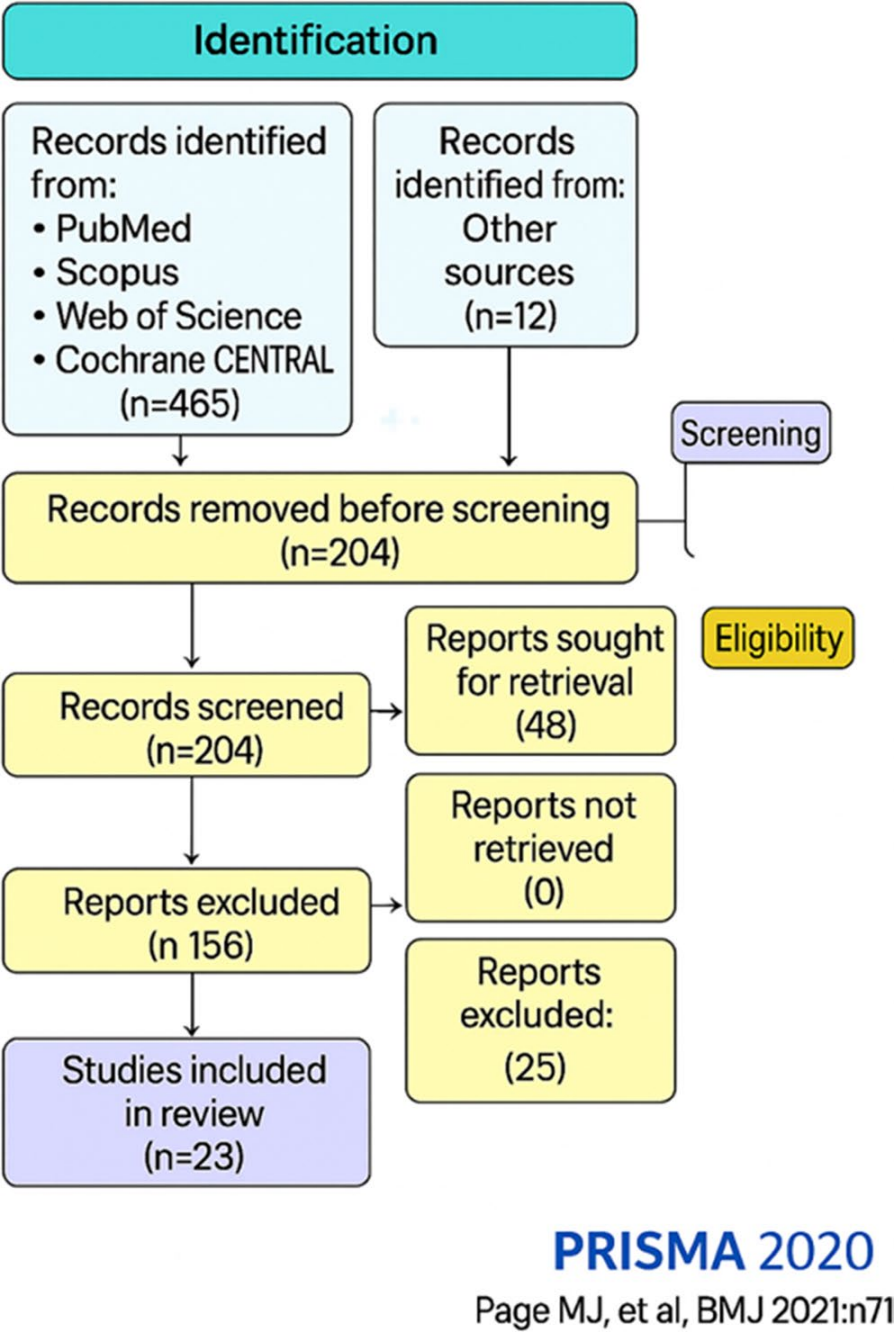
PRISMA 2020 flow diagram for study selection

A total of 465 records were identified through database searching (PubMed [MEDLINE], Scopus, Web of Science, Cochrane CENTRAL) and 12 records through other sources. After removing 173 duplicates, 204 records remained for title/abstract screening. We excluded 156 records at screening. Forty-eight full-text articles were assessed for eligibility; 25 were excluded. Twenty-three studies met the inclusion criteria and were included in the review (Fig. 1).

### Characteristics of the studies

The twenty-three observational studies were published between 2011 and 2024, encompassing 2,689 patients with perforated diverticulitis and free intraperitoneal air who were intended to be treated with conservative medical treatment (NOM – Non-Operative Management) (Table 1).

**Table 1:** Characteristics of the included studies

First author and year of publication	Country	Type of study	Time of enrollement	Follow-up period (months)
Dharmarajan et al. 2011 [6]	USA	R	1995–2008	21,2 (median)
Costi et al. 2012 [7]	France	R	2001–2010	71
Lahat et al. 2013 [8]	Israel	P	2000–2006	88 (median)
Bridoux et al. 2014 [9]	France	R	2008–2012	12
Mora Lopez et al. 2013 [10]	Spain	P	2010–2013	1
Sallinen et al. 2014 [11]	Finlandia	R	2006–2010	n.r
Thorisson et al. 2016 [12]	Sweden, Iceland	P	2003–2010	12
Colas et al. 2017 [13]	Francia	R	2009–2015	-
Titos-García et al. 2017 [14]	Spain	R	2010–2015	n.r.
You et al. 2018 [15]	USA	RCT	-	-
Thorisson et al. 2018 [16]	Sweden	R	2010–2014	-
Bolkenstein et al. 2019 [17]	Netherlands	R	2005–2017	11
Meyer et al. 2019 [18]	Switzerland	R	2005–2009	120
Fernandez et al., 2020 [19]	Chile	R	2009–2015	71
Dissanayake et al. 2020 [20]	Australia	R	2016–2019	n.r.
Vogels et al. 2020 [21]	Netherlands	R	2016–2018	n.r.
Puerta et al., 2021 [22]	Spain	R	2007–2018	n.r.
Sebastian-Tomas et al., 2022 [23]	Spain	P	2018–2019	12
Martín-Román et al. 2022 [24]	Spain	R	2015–2019	10.3
Ocana et al., 2023 [25]	Italy	R	2016/2020	-
Tejedor et al. 2023 [26]	Spain	R	2015–2019	-
Di Fratta et al. 2022 [27]	Europa + South America	P	2020–2021	12
Aubert et al. 2024 [28]	France	R	2015–2021	21

*R* retrospectives studies, *P* prospective studies, *RCT* randomized control trial

Seventeen of these studies are retrospective [6, 7, 9, 11, 13, 14, 16–25, 28], fifth are prospective [8, 10, 12, 23, 26], and one is a randomized control trial [15]. One study [16] utilized data from the multicentric randomized AVOD study, which involved ten centers in Sweden and one in Iceland between October 2003 and January 2010. Additionally, one study integrated patient data with that from the DIABOLO trial [17], a randomized controlled study comparing antibiotic treatment with non-antibiotic treatment in patients with uncomplicated diverticulitis. Fifth studies are multicentric [12, 13, 21, 25, 26], while two included a control group [18, 26].

These studies were conducted globally: one in Australia [20], two in the USA [6, 15], one in South America (Chile) [19], and one in Israel [8]. The other studies were carried out across various European countries: Finland [11], France [7, 9, 13, 28], Iceland/Sweden [12], the Netherlands [17, 21], Spain [10, 14, 22–25], and Switzerland [18]. Another study was conducted between Europe and South Africa [26].

The patient enrollment periods in these studies ranged from a minimum of 1 year to a maximum of 13 years, resulting in a total observation period extending approximately 30 years, from 1995 to 2023.

The median age of NOM patients varied between 51 [14] and 65 years [18]. Perioperative risk was evaluated using the Charlson Index in one study [20] and the ASA in four studies [9, 14, 24, 28]. Among NOM patients, ASA I was most common comorbidity (41–60%) [9, 17] followed by ASA II (35–44%) [7, 17]. The rate of previous episodes of acute diverticulitis ranged from 2% [26]to 19% [11].

All studies included patients with acute diverticulitis of the colon, though the specific location (left colon and sigmoid colon) was only reported in five studies [9, 12, 18, 23, 28]. Pneumoperitoneum rates were reported in all studies, with distinctions made between pericolic extraluminal air and distant free air (Table 2). Definitions of pericolic extraluminal air varied, with some studies defining it as air within 5 cm of the inflamed intestinal segment as per WSES guidelines. Rates for pericolic air ranged from 79% [9] to 100% [26]; our systematic review found a rate of 75% (2,021 patients). Rates for distant air varied from 15% [14] to 100% [25]; our systematic review recorded a rate of 24% (643 patients). Only three studies reported distant air in the retroperitoneal space, with a rate of 1% [11, 15, 28].

**Table 2:** Locations of free air in patients who underwent Non-Operative management (NOM)

First author and year of publication	Overall NOM population	Only pericolic air	Distant air	Only retroperitoneal air
Dharmarajan et al. 2011 [6]	46	19	27	NR
Costi et al. 2012 [7]	39	31	8	NR
Lahat et al. 2013 [8]	23	23	0	NR
Bridoux et al. 2014 [9]	66	56	10	NR
Mora Lopez et al. 2013 [10]	14	14	0	NR
Sallinen et al. 2014 [11]	125	82	29	14
Thorisson et al. 2016 [12]	27	26	1	NR
Colas et al. 2017 [13]	91	34	57	NR
Titos-García et al. 2017 [14]	64	51	13	NR
You et al. 2018 [15]	116	107	0	9
Thorisson et al. 2018 [16]	107	95	12	NR
Bolkenstein et al. 2019 [17]	109	109	0	NR
Meyer et al. 2019 [18]	56	56	0	NR
Fernandez et al., 2020 [19]	37	29	8	NR
Dissanayake et al. 2020 [20]	43	32	11	NR
Vogels et al. 2020 [21]	101	101	Nr	NR
Puerta et al., 2021 [22]	11	11	0	NR
Sebastian-Tomas et al., 2022 [23]	128	122	6	NR
Martín-Román et al. 2022 [24]	80	65	15	NR
Ocana et al., 2023 [25]	369	Nr	369	NR
Tejedor et al. 2023 [26]	744	744	Nr	NR
Di Fratta et al. 2022 [27]	198	142	56	NR
Aubert et al. 2024 [28]	95	72	21	2
Total	2689	2.021/2.689 (75.16%)	643/2.689 (23.91%)	25/2.689 (0.93%)

Initial treatment for patients with acute diverticulitis and pericolic extraluminal air in all studies was conservative, involving dietary restrictions, fluid therapy, and antibiotics. In three studies, some patients did not receive any antibiotics [12, 16, 21]. Predominant antibiotics used included fluoroquinolones, aminoglycosides, second and third-generation cephalosporins, metronidazole, carbapenems, piperacillin-tazobactam, and penicillins such as ampicillin or amoxicillin ± clavulanic acid. Eight studies specified the type of antibiotics used [7, 9–12, 18, 20, 23, 26, 28].

Follow-up data was reported in eleven studies [6–9, 12, 16–19, 23, 24, 26, 28], which is crucial for assessing long-term outcomes and treatment effectiveness.

We included 2,689 patients intended for conservative treatment (overall population). For individual outcomes the denominators differ because not all studies reported all outcomes.

- Percutaneous drainage: PD use was reported in 15 studies including 1,920 patients; PD was performed in 138/1,920 patients (7.2%). Studies that did not report PD use were excluded from this denominator.
- Mortality: in-hospital mortality data were available from 18 studies including 2,223 patients; total in-hospital deaths were 23, giving an observed mortality of 23/2,223 (1.03%). Studies that did not report mortality were excluded from the mortality denominator.
- Surgical failure and procedures: the count of patients undergoing emergency surgery (*n* = 284) and the detailed breakdown of surgical procedures are calculated on the subset of studies that reported procedures and reasons for surgery; denominators for individual procedure types are specified in Table 2.

### Assessment of risk of bias in included studies

**Fig. 2:**
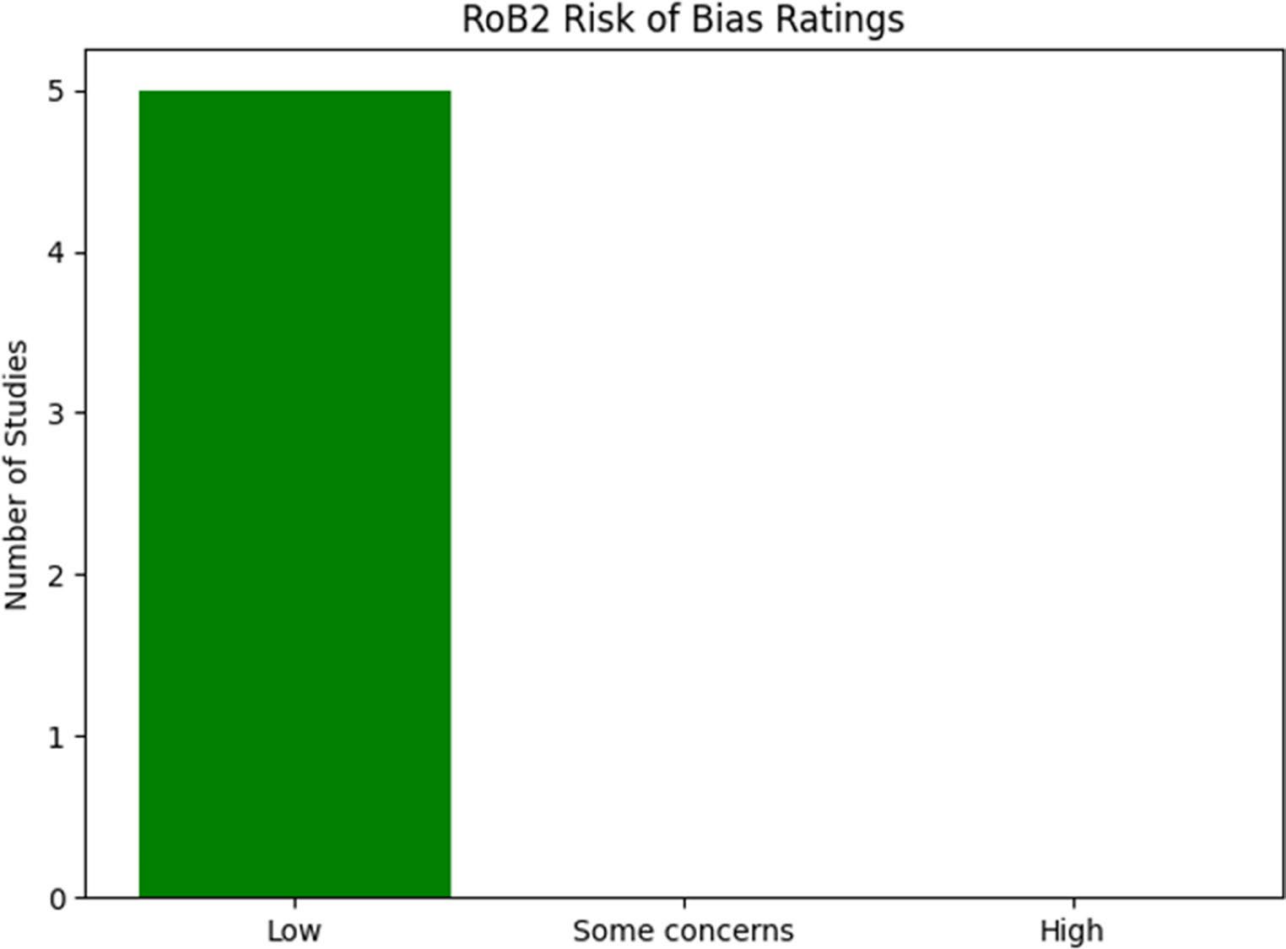
Risk of bias domains for included studies RCTs using the Rob2

**Fig. 3:**
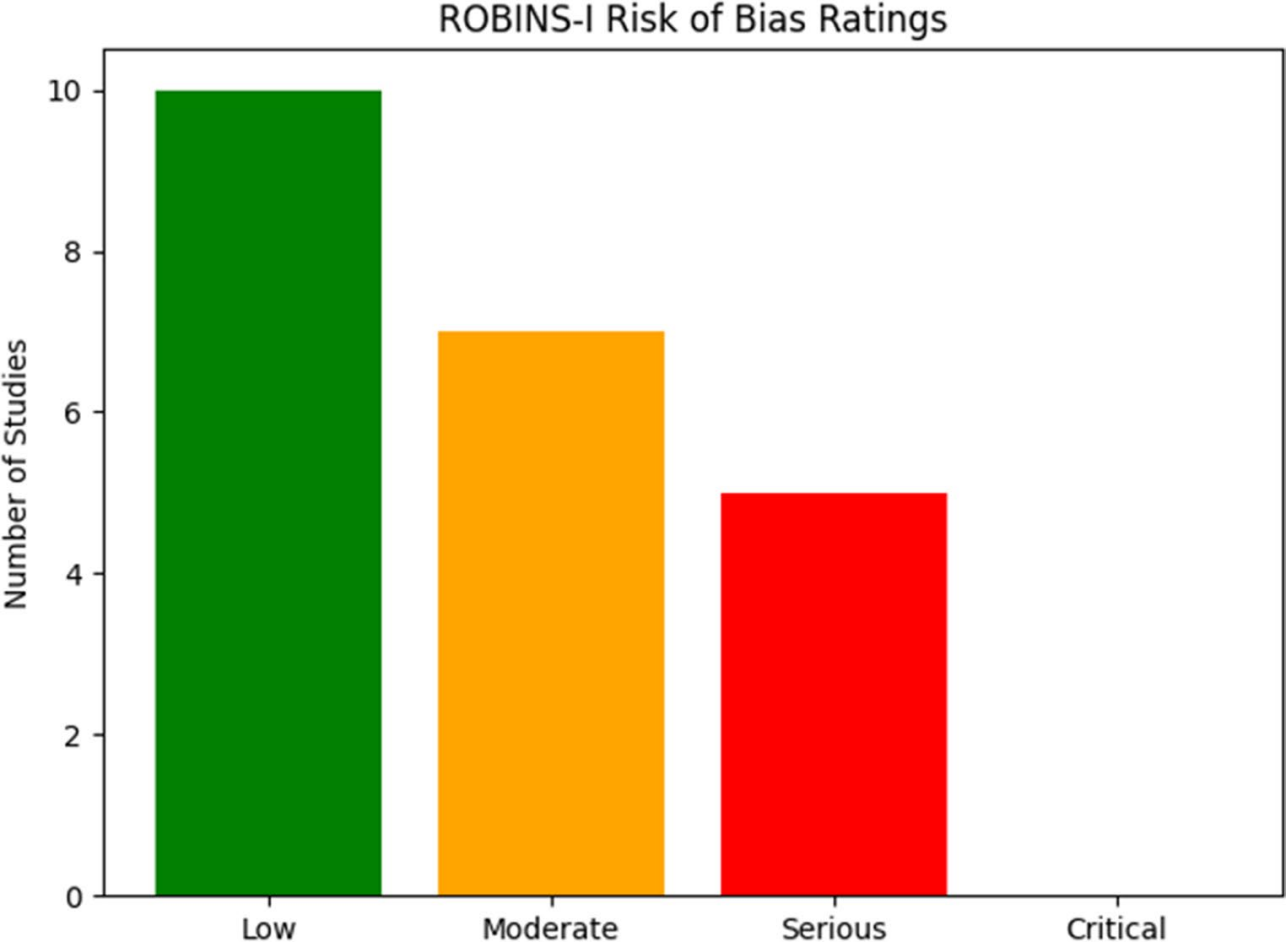
Risk of bias domains for included observational studies using the Rob1

We assessed risk of bias for all included studies: the single randomized trial [15] was evaluated with RoB 2 and judged at low overall risk of bias across RoB 2 domains. The 22 non-randomized studies were evaluated with ROBINS-I: most observational studies had at least one domain with Moderate or Serious risk of bias, most commonly in participant selection, missing data, and measurement of outcomes. Five studies were rated Serious in selection bias and six studies had serious concern for missing data. A comprehensive study-level ROBINS-I and RoB 2 is provided in following Figs. 2 and 3.

### Results of meta-analysis

**Fig. 4:**
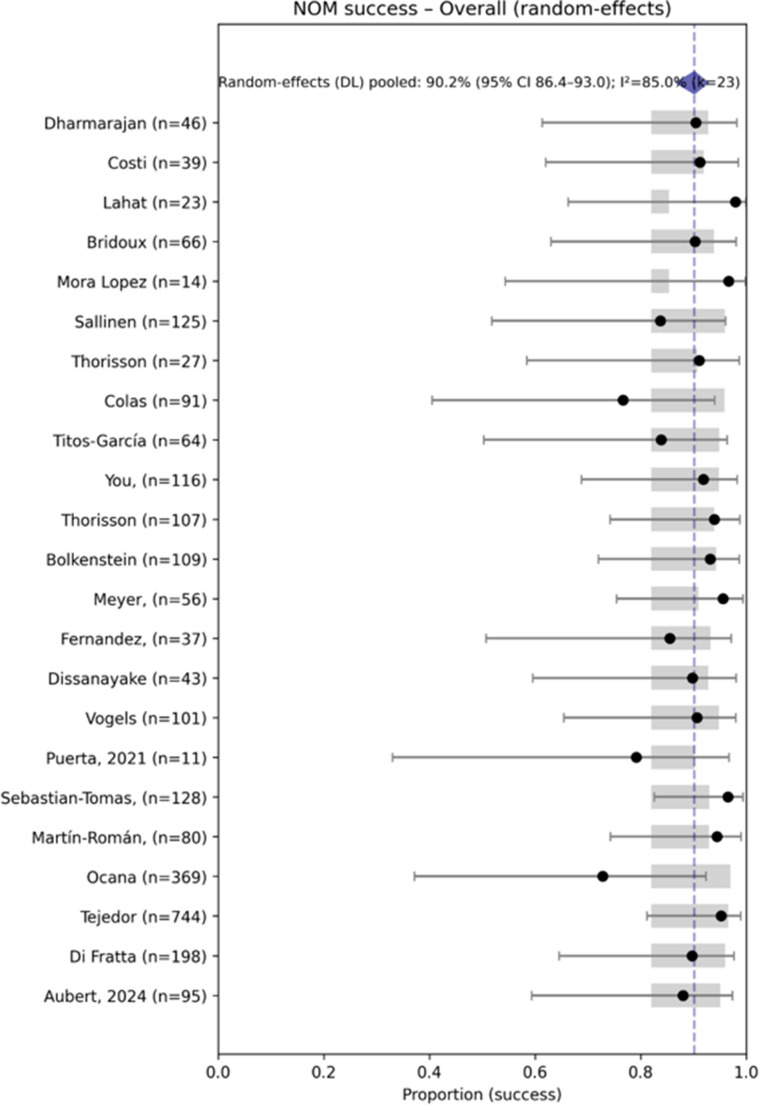
Forest plot: Overall NOM success (random-effects)

In patients with free peritoneal air resulting from perforated diverticulitis, the pooled probability of successful non-operative management (NOM) across 2,689 patients was 90.2% (95% CI 86.4–93) (Fig. 4), with high heterogeneity (I² = 85%).

Sensitivity analyses excluding the largest studies and restricting to prospective cohorts produced estimates consistent with the primary analyses. Stratification by failure definition revealed that studies counting percutaneous drainage (PD) as a failure (Table 3) reported substantially higher PD utilization (mean 23.3%) compared with studies that considered PD part of successful NOM (mean 5.5%), identifying inconsistent failure definitions as an important source of heterogeneity (Table 4).

**Table 3:** Success of Non-Operative management (NOM) in patients with free air

First author and year of publication	NOM population (number of patients)	Successful NOM		Failure of NOM (number of patients)
		Number of patients	Rate (%)	
Dharmarajan et al. 2011 [6]	46	42	91.3	4
Costi et al. 2012 [7]	39	36	92.3	3
Lahat et al. 2013 [8]	23	23	100	0
Bridoux et al. 2014 [9]	66	60	90.9	6
Mora Lopez et al. 2013 [10]	14	14	100	0
Sallinen et al. 2014 [11]	125	105	84	20
Thorisson et al. 2016 [12]	27	25	92.6	2
Colas et al. 2017 [13]	91	70	76.9	21
Titos-García et al. 2017 [14]	64	54	84.4	10
You et al. 2018 [15]	116	107	92.2	9
Thorisson et al. 2018 [16]	107	101	96.4	6
Bolkenstein et al. 2019 [17]	109	102	93.6	7
Meyer et al. 2019 [18]	56	54	96.4	2
Fernandez et al., 2020 [19]	37	32	86.5	5
Dissanayake et al. 2020 [20]	43	39	90.7	4
Vogels et al. 2020 [21]	101	92	91.1	9
Puerta et al., 2021 [22]	11	9	81.8	2
Sebastian-Tomas et al., 2022 [23]	128	124	96.9	4
Martín-Román et al. 2022 [24]	80	76	95	4
Ocana et al., 2023 [25]	369	269	72.9	100
Tejedor et al. 2023 [26]	744	709	95.3	35
Di Fratta et al. 2022 [27]	198	178	89.9	20
Aubert et al. 2024 [28]	95	84	88.4	11
Total	2.689	2.405/2.689 (89.44)		284/2.689 (10.56)

Table 2: Locations of free air in patients who underwent NOM.

**Table 4:** Definition of failure and percutaneous drainage (PD) utilization in included studies. Column headings: author and year | definition of failure | PD utilization (n) | PD rate (%). NR = not reported

Author and Year	Definition of Failure	PD Utilization (*n*)	PD Rate (%)
Dharmarajan et al. 2011 [6]	Emergency surgery or PD	18	39.1%
Costi et al. 2012 [7]	Emergency surgery or PD	3	10.3%
Lahat et al. 2013 [8]	Emergency surgery only	4	17.4%
Bridoux et al. 2014 [9]	Emergency surgery or PD	9	13.6%
Mora Lopez et al. 2013 [10]	Emergency surgery only	3	21.4%
Sallinen et al. 2014 [11]	Emergency surgery or PD	NR	NR
Thorisson et al. 2016 [12]	Emergency surgery only	NR	NR
Colas et al. 2017 [13]	Surgery, radiologic drainage or death	8	8.8%
Titos-García et al. 2017 [14]	Emergency surgery or PD	4	6.3%
You et al. 2018 [15]	Emergency surgery only	30	25.9%
Thorisson et al. 2018 [16]	Emergency surgery only	11	10.3%
Bolkenstein et al. 2019 [17]	Emergency surgery only	2	1.8%
Meyer et al. 2019 [18]	Emergency surgery only	NR	NR
Fernandez et al., 2020 [19]	Emergency surgery only	NR	NR
Dissanayake et al. 2020 [20]	Emergency surgery only	NR	NR
Vogels et al. 2020 [21]	Emergency surgery only	NR	NR
Puerta et al., 2021 [22]	Emergency surgery only	NR	NR
Sebastian-Tomas et al., 2022 [23]	Emergency surgery; PD = success	3	2.3%
Martín-Román et al. 2022 [24]	Emergency surgery; PD = success	3	3.8%
Ocana et al., 2023 [25]	Emergency surgery; PD = success	NR	NR
Tejedor et al. 2023 [26]	Emergency surgery; PD = success	12	1.6%
Di Fratta et al. 2022 [27]	Emergency surgery; PD = success	23	11.6%
Aubert et al. 2024 [28]	Emergency surgery; PD = success	5	5.3%
Total	—	**138/1920**	**7.2%**

Urgent surgery is required when conservative medical treatment fails due to sepsis, clinical deterioration, or peritonitis. Additionally, in some studies, the use of percutaneous drainage (PD) is considered a failure of NOM [8, 10, 13, 17, 19, 21].

A subgroup analysis demonstrated a substantial difference in PD utilization between groups. Group A (PD = failure) had a mean PD rate of 23.3%; Group B (PD ≠ failure) had a mean PD rate of 5.5%. The overall pooled PD rate across all studies was 7.2%.

The large difference in PD rates between Group A and Group B shows that inconsistent operational definitions of “failure” introduce marked heterogeneity. When PD is classified as a failure, studies report higher failure rates and greater PD utilization; when PD is considered part of successful conservative management, the same interventions increase reported success. This definitional variability can bias pooled estimates of NOM success and reduce comparability across cohorts.

### Implications and limitations

- We report pooled estimates alongside subgroup‑specific summaries stratified by the operational definition of failure; therefore, overall pooled success rates should be interpreted with caution. The subgroup analyses are essentially descriptive because not all studies reported percutaneous drainage (PD) data and several did not explicitly state their failure definition. These missing data likely affected subgroup denominators and reduced the precision of our estimates.Residual heterogeneity may remain despite stratification, and the variability in how “failure” is defined across studies is a key limitation that lowers confidence in the pooled success rates. This definitional heterogeneity can introduce bias into pooled estimates and complicate comparisons between studies.

- Recommendation: future primary studies should adopt a standardized, explicit definition of failure that clearly states whether PD is counted as a failure event or considered part of successful non‑operative management (NOM). Consistent definitions willi improve comparability, reduce bias, and increase the certainty of pooled estimates.

### Comparative analysis of NOM success according to air location

**Fig. 5:**
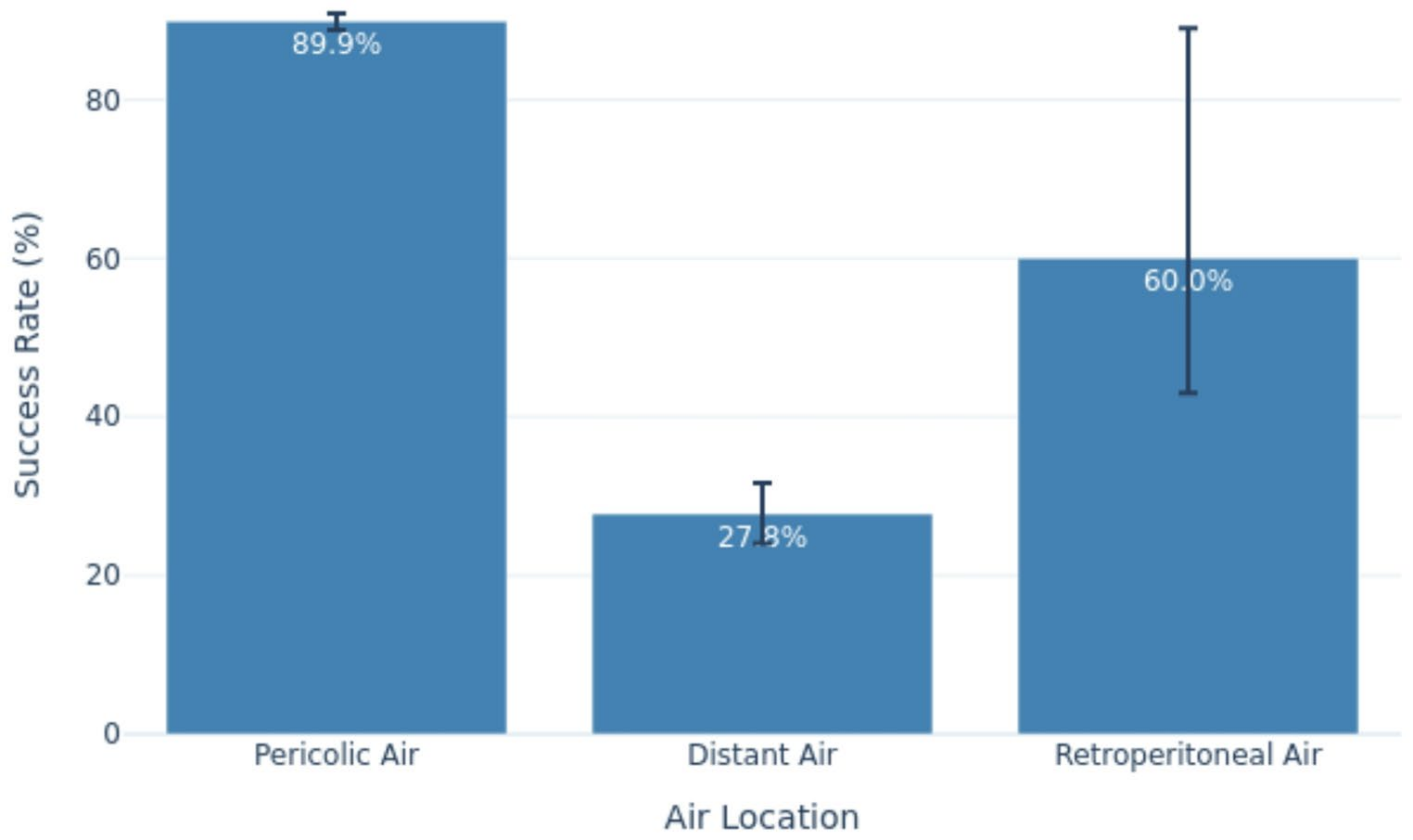
Success rates of non-operative management (NOM) in patients with perforated diverticulitis according to the anatomical location of free air

 Significant differences in NOM success rates were observed according to the anatomical location of extraluminal air (Table 5, Fig. 5).

Among 2,021 patients with pericolic air, the success rate of NOM was reported in 1,816 patients: 89.9%.

**Table 5:** Success of Non-Operative management (NOM) in different air anatomical locations

First author and year of publication	Succesfull NOM for pericolic air		Successful NOM for distant air		Successful NOM for retroperitoneal air	
	Number of patients	Rate (%)	Number of patients	Rate (%)	Number of patients	Rate (%)
Dharmarajan et al. 2011 [6]	17	89.5	25	92.6	Nr	Nr
Costi et al. 2012 [7]	28	90.3	8	100	Nr	Nr
Lahat et al. 2013 [8]	23	100	Nr	Nr	Nr	Nr
Bridoux et al. 2014 [9]	Nr	Nr	Nr	Nr	Nr	Nr
Mora Lopez et al. 2013 [10]	14	100	Nr	Nr	Nr	Nr
Sallinen et al. 2014 [11]	81	98.8	18	37.9	6	42.8
Thorisson et al. 2016 [12]	Nr	Nr	Nr	Nr	Nr	Nr
Colas et al. 2017 [13]	29	85.3	43	24.6	Nr	Nr
Titos-García et al. 2017 [14]	46	90.2	8	38.5	Nr	Nr
You et al. 2018 [15]	99	92.5	Nr	Nr	8	88.8%
Thorisson et al. 2018 [16]	90	94.7	Nr	Nr	Nr	Nr
Bolkenstein et al. 2019 [17]	102	93.6	Nr	Nr	Nr	Nr
Meyer et al. 2019 [18]	54	96.4	Nr	Nr	Nr	Nr
Fernandez et al., 2020 [19]	Nr	Nr	Nr	Nr	Nr	Nr
Dissanayake et al. 2020 [20]	30	93.8	9	18.2	Nr	Nr
Vogels et al. 2020 [21]	92	91.1	Nr	Nr	Nr	Nr
Puerta et al., 2021 [22]	9	81.8	Nr	Nr	Nr	Nr
Sebastian-Tomas et al., 2022 [23]	118	96.7	6	100	Nr	Nr
Martín-Román et al. 2022 [24]	64	98.5	12	20	Nr	Nr
Ocana et al., 2023 [25]	Nr	Nr	Nr	Nr	Nr	Nr
Tejedor et al. 2023 [26]	709	95.3	Nr	Nr	Nr	Nr
Di Fratta et al. 2022 [27]	142	100	36	35.7	Nr	Nr
Aubert et al. 2024 [28]	69	95.8	14	33.3	1	50%
Total	1.816/2.021	89,9%	179/643	27.83	15/25	60%

Among 643 patients with distant air, the success rate of NOM was reported in 179 patients: 27.8%.

The limited number of studies on retroperitoneal free air reported NOM success rates ranging from 43% [11] to 89% [15].

In patients with unsuccessful NOM, surgical intervention was reported in 144 cases (Table 6). The most common procedure was Hartmann’s procedure (mean 6.5% ± SD 5.484), followed by resection and anastomosis with covering stoma (ileostomy) (mean 4.5% ± SD 3.882), and laparoscopic lavage (mean 2.3% ± SD 1.00).

**Table 6:** Type of surgical treatment in failure of NOM

First author	Hartmann reverse procedure	Resection and anastomosis +/- ileostomy	Peritoneal lavage	Other
Dharmarajan et al. 2011 [6]	-	-	199	-
Costi et al. 2012 [7]	0	1	2	0
Lahat et al. 2013 [8]	-	-	-	-
Bridoux et al. 2014 [9]	6	3	0	0
Mora Lopez et al. 2013 [10]	-	-	-	-
Sallinen et al. 2014 [11]	14	3	1	0
Thorisson et al. 2016 [12]	-	-	-	-
Colas et al. 2017 [13]	10	3	7	0
Titos-García et al. 2017 [14]	0	8	1	0
You et al. 2018 [15]	-	-	-	-
Thorrison et al. 2018 [16]	-	-	-	-
Bolkenstein et al. 2019 [17]	2	4	1	0
Meyer et al. 2019 [18]	-	-	-	-
Fernandez et al. 2020 [19]	-	-	-	-
Dissanayake et al. 2020 [20]	7	0	0	0
Puenta et al. 2021 [21]	-	-	-	-
Vogels et al. 2020 [22]	5	1	1	2
Sebastian-Tomas et al. 2022 [23]	-	-	-	-
Martín-Román et al. 2022 [24]	-	-	-	-
Ocana et al. 2023 [25]	-	-	-	-
Tejedor et al. 2023 [26]	17	10	8	0
Di Fratta et al. 2022 [27]	8	12	-	-
Aubert et al. 2024 [28]	3	4	-	-
TOTAL	72	49	21	2

Data regarding the use of percutaneous drains were reported in 1,920 patients enrolled in 15 studies [6–10, 13–17, 23, 24, 26–28] (Table 7). Percutaneous drainage of abscesses was performed in 7.2% (138 patients) (mean 12% ± SD 10.29): 2% in patients with pericolic air (mean 3.2% ± SD 2.92) and 30% in those with distant free air (mean 24.3% ± 18.58).

**Table 7:** Use of percutaneous drainage (PD) for concomitant abscesses

First author and year of publication	PD utilization in NOM	
	Number of patients	Rate of PD
Dharmarajan et al. 2011 [6]	18	39.1%
Costi et al. 2012 [7]	3	10.3%
Lahat et al. 2013 [8]	4	17.4%
Bridoux et al. 2014 [9]	9	13.6%
Mora Lopez et al. 2013 [10]	3	21.4%
Sallinen et al. 2014 [11]	Nr	Nr
Thorisson et al. 2016 [12]	Nr	Nr
Colas et al. 2017 [13]	8	8.8%
Titos-García et al. 2017 [14]	4	6.3%
You et al. 2018 [15]	30	25.9%
Thorisson et al. 2018 [16]	11	10,3%
Bolkenstein et al. 2019 [17]	2	1.8%
Meyer et al. 2019 [18]	Nr	Nr
Fernandez et al., 2020 [19]	Nr	Nr
Dissanayake et al. 2020 [20]	Nr	Nr
Vogels et al. 2020 [21]	Nr	Nr
Puerta et al., 2021 [22]	Nr	Nr
Sebastian-Tomas et al., 2022 [23]	3	2.3%
Martín-Román et al. 2022 [24]	3	3.8%
Ocana et al., 2023 [25]	Nr	Nr
Tejedor et al. 2023 [26]	12	1.6%
Di Fratta et al. 2022 [27]	23	11.6%
Aubert et al. 2024 [28]	5	5,3%
Total	138/1.920	7.2%

Patients undergoing NOM exhibited a low mortality rate following initial conservative treatment, at 1.03% (23 patients) (Table 8).

**Table 8:** Hospital mortality in patients underwent NOM

First author and year of publication	Mortality
Dharmarajan et al. 2011 [6]	3
Costi et al. 2012 [7]	0
Lahat et al. 2013 [8]	2
Bridoux et al. 2014 [9]	1
Mora Lopez et al. 2013 [10]	-
Sallinen et al. 2014 [11]	1
Thorisson et al. 2016 [12]	-
(Colas et al. 2017) [13]	1
Titos-García et al. 2017 [14]	1
You et al. 2018 [15]	-
Thorisson et al. 2018 [16]	5
Bolkenstein et al. 2019 [17]	1
Meyer et al. 2019 [18]	-
Fernandez et al., 2020 [19]	1
Dissanayake et al. 2020 [20]	0
Vogels et al. 2020 [21]	0
Puerta et al., 2021 [22]	1
Sebastian-Tomas et al., 2022 [23]	-
Martín-Román et al. 2022 [24]	2
Ocana et al., 2023 [25]	-
Tejedor et al. 2023 [26]	2
Di Fratta et al. 2022 [27]	0
Aubert et al. 2024 [28]	2
TOTAL	23/2.223 (1,03%)

## Heterogeneity and Sensitivity Analysis

To address potential sources of heterogeneity, we performed predefined sensitivity and subgroup analyses. Sensitivity analyses were conducted by excluding the largest studies (Tejedor et al., Ocana et al.) and restricting the dataset to prospective cohorts only. These analyses yielded pooled estimates consistent with the primary analysis, confirming the robustness of our findings. Specifically, the success rate of non-operative management (NOM) remained above 88% in both scenarios.

Subgroup analysis by study design showed that prospective studies (*n* = 5) reported a pooled NOM success rate of 91.2% (95% CI: 88.1–93.7), comparable to retrospective studies. This suggests that study design did not significantly influence the overall outcome.

## Discussion

Approximately 15% of patients with acute diverticulitis exhibit pericolic extraluminal air, typically diagnosed by CT scan, which influences treatment decisions. The optimal management approach remains debated, with limited guidelines available, except for those updated by the World Society of Emergency Surgery (WSES) in 2020 [1]. According to WSES guidelines published in 2020, patients with CT findings of pericolic air could be approached with a non-operative treatment with antibiotic therapies (weak recommendation based on low quality evidence, 2 C), while the non-operative management of patients with CT findings of distant air and without diffuse intra-abdominal fluid could be considered only if a close follow-up could be performed (weak recommendation based on low quality evidence, 2 C).

In our analysis, the pooled estimates indicate that NOM is effective in the large majority of patients with pericolic free air (≈ 90%), while success is substantially lower in patients with distant free air (≈ 28%). The moderate to high heterogeneity—particularly high in the distant air subgroup—reflects clinical and methodological variability across studies, including differences in patient selection, imaging definitions, timing and type of interventions, and operational definitions of treatment “failure” (notably whether percutaneous drainage was counted as failure). Furthermore, the outcome denominators vary because reporting across primary studies was incomplete and heterogeneous. To avoid biased imputations we used study-specific denominators for each outcome (complete-case analysis): each pooled estimate includes only patients from studies that explicitly reported the relevant outcome.

Predictive factors for the failure of conservative treatment were identified in most of the studies reviewed. The primary distinction between the successful group and those requiring emergency surgery was the presence of free fluid in the pelvis and distant free air. Additionally, laboratory values indicating inflammation and infection were significant. Although all authors mentioned clinical factors, they were not statistically significant in determining the prognosis of conservative treatment. This finding underscores the importance of staging with a contrast-enhanced CT scan for all patients suspected of having acute diverticulitis.

The major challenge in these patients is decision-making regarding the feasibility of conservative treatment for those with distant free air, as its effectiveness remains controversial. Our systematic review indicates that the success rate was significantly higher in patients with pericolic compared to those with distant air, consistent with the latest WSES guidelines [1].

Furthermore, in this review was reported a limited use of percutaneous drainage (7%), in fact the drainage was performed only for abscess/collection of fluid, not free air.

This review acknowledges that the relatively small number of patients with distant free air may influence the success rates. This might indicate a selection bias where the included studies focused on patients with hemodynamic stability, whereas patients with distant free intra-abdominal perforations often present in conditions necessitating emergency surgery.

Our findings are consistent with the 2020 WSES guidelines [1], which recommend non-operative management (NOM) for patients with pericolic free air and no signs of generalized peritonitis (weak recommendation, low-quality evidence), while advising extreme caution for cases with distant free air, limiting NOM to settings with close clinical and radiological monitoring. However, our review adds a critical quantitative perspective: the pooled success rate of NOM in patients with distant free air is only 27.8%, despite probable selection bias toward hemodynamically stable patients, whereas success in pericolic air cases approaches 90%. This substantial difference reinforces the need for early surgical intervention in distant free air cases, going beyond the general caution suggested by WSES. Our findings align with previous systematic reviews. Compared with previous systematic reviews, our results highlight important differences. van Dijk et al. reported a 94% success rate for pericolic air, slightly higher than our 89.9%, likely due to stricter inclusion criteria and the absence of subgroup analyses based on failure definitions [31]. Similarly, our success rates for conservative treatment, categorized by the total population, pericolic air, and distant air, were comparable to the reviews by Chua [32] and Karentzos [33]. Chua et al. [32] and Karentzos et al. [33] reported overall success rates of 85% and 94.9%, respectively, but neither review clearly distinguished outcomes by air location nor addressed heterogeneity introduced by variable definitions of “failure” (e.g., whether percutaneous drainage was considered failure). Our study not only confirms the safety of NOM in pericolic air but also demonstrates the marked heterogeneity and low reliability of data for distant air, providing subgroup and sensitivity analyses that were lacking in prior reviews. In summary, our work strengthens the evidence base for WSES recommendations and underscores the urgent need for prospective studies with standardized definitions and rigorous methodology. Our review has several important limitations. Only one randomized controlled trial was identified, while the bulk of the evidence derives from observational, mostly retrospective studies, which reduces data homogeneity and limits causal inference. Included studies differed in inclusion criteria, follow‑up duration, definitions of treatment failure and the types of conservative therapy employed, and data were collected over a long period (1995–2022) during which diagnostic practices and guidelines—notably the first WSES recommendations in 2016—evolved [1]. We were also unable to evaluate some potentially relevant risk factors consistently across studies (for example, ASA score). Outcome reporting was heterogeneous, producing different denominators for different endpoints (e.g., PD use: n = 1,920 from 15 studies; mortality: n = 2,223 from 18 studies), a pattern of selective reporting that reduces precision and may introduce bias. Risk‑of‑bias assessments further limit confidence in the results: most studies were observational and several were judged to have Moderate to Serious concerns in domains such as participant selection, missing data and outcome measurement. Although we used a random‑effects meta‑analytic approach to account for between‑study heterogeneity and performed sensitivity analyses excluding higher‑risk studies, residual bias may still have affected pooled estimates. For these reasons, pooled proportions should be interpreted cautiously and in the context of the study‑level ROBINS‑I and RoB 2 assessments provided.

## Conclusions

Non-operative management (NOM) may be a feasible option for carefully selected, hemodynamically stable patients with acute diverticulitis and pericolic extraluminal air. However, the overall quality of evidence remains low, with most included studies being observational and retrospective, and significant heterogeneity in patient selection, definitions of treatment failure, and outcome reporting.

In patients with distant free air, the pooled success rate of NOM was markedly lower (27.8%), despite a probable selection bias favoring more stable patients. These findings suggest that early surgical management should be strongly considered for patients with distant free air, given the high failure rate of conservative treatment. If a non-operative approach is pursued, it should be limited to highly selected cases under strict clinical and radiological monitoring, with a low threshold for surgical intervention.

Future prospective studies with standardized definitions and rigorous methodology are needed to better define the role of NOM in patients with perforated diverticulitis and distant free air.

## Data Availability

The data used to support the finding of this study are included within the article. The data presented in this study are available on request.

## Declarations

### **Research registration unique identifying number (UIN) **

Our study is a systematic review of literature: so the research registration unique identifying number is not necessary.
